# A dataset cataloging product-specific human appropriation of net primary production (HANPP) in US counties

**DOI:** 10.1016/j.dib.2023.109530

**Published:** 2023-08-31

**Authors:** Suman Paudel, Kaeli Mueller, Gustavo Ovando-Montejo, Lauren Tango, Richard Rushforth, Christopher Lant

**Affiliations:** a Department of Environment and Society, Quinney College of Natural Resources, Utah State University, 5215 Old Main Hill, Logan, UT 84322-5215, USA; b Utah State University-Blanding 576 West 200 South, Blanding, UT, USA; c School of Informatics, Computing and Cyber Systems, Northern Arizona University, Flagstaff, AZ 86011, USA; d Utah Agricultural Experiment Station, USA

**Keywords:** Ecological footprint, Human appropriation of net primary production, Social ecology, Social metabolism, United States

## Abstract

This paper describes the dataset associated with the paper “Product-Specific Human Appropriation of Net Primary Production (HANPP) in US Counties” (Paudel et al., 2023). This dataset comprises human appropriation of net primary production (HANPP) values for 3101 counties in the conterminous US for the years 1997, 2002, 2007, and 2012. For this dataset, HANPP is the carbon content of specific crop, timber, and livestock grazing products appropriated by humans in a county in a year. To calculate HANPP, raw agricultural data were downloaded from public databases such as USDA-National Agricultural Statistics Service Quick Stats and Cropland Data Layer, US Forest Service Timber Product Output, and NPP data from MODIS. These data were processed in Microsoft Excel using stoichiometry derived from established scientific literature. HANPP was partitioned by year, county, product, used and unused and above- and below-ground. This complete dataset is published in Mendeley Data and the methods used to compile them are included to make our research well documented, reproducible, and useful for future studies.

Specifications Table

**Table:** 

Subject	Environmental science Ecological modeling Human appropriation of net primary production
Specific subject area	The subject area is the 48 states of the conterminous United States with data observations at the county level. Availability of data on crop production, timber cutting, and livestock grazing is the criterion used to identify the spatial and temporal framework for the study.
Type of data	Tables (Microsoft Excel), R markdown file
How the data were acquired	Raw data were acquired by downloading them from public databases for the available years (1997, 2002, 2007, 2012) for each of 3101 counties in the conterminous U.S. Net primary production data were acquired from MODIS and Landsat satellite imagery as raster data. Crop yield data are from USDA National Agricultural Statistics Service Quick Stats and Cropland Data Layer databases in spreadsheet and raster form, respectively. Timber HANPP data were acquired from US Forest Service databases as spreadsheets. For grazing HANPP, cattle inventory data were acquired from Quick Stats in spreadsheet form and grazing allotment data from US Forest Service and Bureau of Land Management in tabular form. Datasets were processed and organized in Microsoft Excel.
Data format	Analyzed Filtered Processed
Description of data collection	Applying stoichiometry estimates from scientific literature to the raw data, HANPP estimates were derived for each product, for each year of study. These are partitioned for used and unused and above and below-ground portions. Each are reported as mass of carbon in kilotonnes and as densities in gCm^−2^yr^−1^ both as county-wide averages and as on-site densities. National totals and means are also included as well as estimates of NPP and NPP(ecological). The repository includes a total of about 3,000,000 measures of HANPP.
Data source location	Source data: MODIS, 2020, Numerical Terradynamic Simulation Group, https://www.umt.edu/numerical-terradynamic-simulation-group/project/landsat/landsat-productivity.php U.S. Department of Interior, 2019. Bureau of Land Management. Rangeland Administration System Reports. Available at: https://reports.blm.gov/reports/ras/. USDA. Forest Service, 2019. Timber Product Output dataset (TPO)/National TPO. USDA-NASS CropScape – Cropland Data Layer. 2012. Available at: https://nassgeodata.gmu.edu/CropScape/ USDA-NASS Quick Stats, 2019, NASS, https://quickstats.nass.usda.gov/
Data accessibility	**Repository name**: A Dataset Cataloging Product-Specific Human Appropriation of Net Primary Production (HANPP) in US Counties Mendeley data repository (https://data.mendeley.com/datasets/ksyd2cr9cr/5) **Data identification number**: 10.17632/ksyd2cr9cr.5 **Direct URL to data**: https://data.mendeley.com/datasets/ksyd2cr9cr/5 **Instructions for accessing these data**: Visit the above URL to access this data repository.
Related research article	Paudel, S., K. Mueller, G. Ovando-Montejo, R. Rushforth, L. Tango, C. Lant, 2023. Product-specific human appropriation of net primary production in U.S. counties, *Ecological Indicators*.150: 110241. doi:10.1016/j.ecolind.2023.110241.

## 1 Value of the Data

• These data are useful because they provide the first product-specific, county-level measurements of HANPP in the U.S., a valid indicator of human land use intensity and an ecological indicator that has advantages over ecological footprint due to its spatial specificity and empirical foundation in net primary production.
• These data will be beneficial to researchers and land managers who want to measure HANPP on a large scale in relation to other environmental variables.
• HANPP can be used in further experiments that look at the effect of human land use on biodiversity, how HANPP can be broken down into different subsets, or how HANPP relates to ecosystem service provision.
• These production-oriented data on HANPP can be paired with data on HANPP consumption and trade to delineate a system of ecological interdependencies.
• HANPP data can be used to describe ‘anthromes’ as human-oriented ecological categories in contrast to biomes.

## 2 Objective

This dataset compiles measurements for 3101 counties in the conterminous U.S. and on a product-specific basis in five-year intervals. The article *Product-Specific Human Appropriation of Net Primary Production (HANPP) in US Counties* [1] is the accompanying paper for this dataset, and it contains background about HANPP and extensive interpretation of the results. This *Data in Brief* article adds to the original research paper by including more details about the data collection, processing, and database structure.

## 3 Data Description

This data repository contains the processed data for HANPP measurements. The HANPP data sets are organized into four folders representing the four years for which HANPP was calculated (1997, 2002, 2007, 2012). Within each year are folders representing the different measurements of HANPP; county-wide HANPP and onsite HANPP density in gCm^−2^yr^−1^ and HANPP mass in kilotonnes C. There are separate folders for the different measurements of HANPP. Within each unit folder are five spreadsheet files that contain the total county HANPP, HANPP partitioned into aboveground and belowground, and HANPP partitioned into used and unused. Note that grazing HANPP is defined as entirely aboveground and entirely used. Note also that we did not calculate onsite HANPP densities for timber because the locations of harvest within a county are undetermined.

File Organization: For each HANPP spreadsheet file, each filename includes the type of HANPP (total, aboveground, belowground, used, or unused), the method of HANPP (county-wide or onsite), the units of HANPP (gCm^−2^yr^−1^ or kilotonnes), and the year (1997, 2002, 2007, or 2012). Each row contains data for a single county. The following list describes the data included in each column:

STATE: The state that the county is in.
STATE_FIPS: The government Federal Information Processing Standard FIPS code for the state.
STATE_COUNTY: The state and county, listed in the following form: ALABAMA_AUTUAGA. This value is unique and can be used as a primary key.
COUNTY: The county name.
COUNTY_FIPS: The government FIPS code for the county.
YEAR: The year that HANPP is being calculated.
COUNTY_AREA: The area of each county in meters squared.
corn_grain: HANPP of corn grain.
corn_silage: HANPP of corn silage.
winter_wheat: HANPP of winter wheat.
spring_wheat_durum: HANPP of durum spring wheat.
spring_wheat_excluding_durum: HANPP of spring wheat other than durum.
soybeans: HANPP of soybeans.
hay_alfalfa: HANPP of alfalfa hay.
cotton_pima: HANPP of pima cotton.
cotton_upland: HANPP of cotton upland.
sorghum: HANPP of sorghum.
other_crops: Average HANPP of other crops that make up the remaining 23% of cropland, after the other top 10 crops.
total_crops: HANPP of all crops, which is the sum of columns “corngrain” through “other_crops”.
hardwood: HANPP of hardwood.
softwood: HANPP of softwood.
total_timber: HANPP of all timber, which is the sum of “hardwood” and “softwood”.
blm_grazing: HANPP of grazing that takes place on Bureau of Land Management land.
usfs_grazing: HANPP of grazing that takes place on US Forest Service land.
private_land_grazing: HANPP of grazing that takes place on private land.
total_grazing: HANPP of all grazing, which is the sum of “blm_grazing,” “usfs_grazing” and “private_land_grazing”.
total_hanpp: The total HANPP for the county, which is the sum of “total_crops,” “total_timber” and “total_grazing.”

Note that a blank cell indicates that there are no data for that particular HANPP product. This can be assumed to constitute negligible HANPP.

Within the data repository, there is also a folder called “totals” of national HANPP totals for the years 1997, 2002, 2007, and 2012. Within this folder are two folders called “HANPP_NPP_NPPeco” (which contains total HANPP(harvest), NPP, and NPP(ecological) measurements) and “national_HANPP” (which contains national totals for HANPP components). The “HANPP_NPP_NPPeco” folder contains two folders for county and national totals. The county totals folder contains three folders for the years 2002, 2007, and 2012 (there is no NPP data available for 1997). Within each of these year-based folders are two spreadsheet files for gCm^−2^yr^−1^ or kilotonnes. Each filename reflects that the totals are for HANPP, NPP, and NPPeco, the scale, the year, and the units. Within the “national HANPP” folder are two folders for the units gCm^−2^yr^−1^ and kilotonnes. Within these unit folders are five spreadsheet files which contain the year totals for specific HANPP products for total HANPP, aboveground HANPP, belowground HANPP, used HANPP and unused HANPP for specific HANPP products.

In the repository there are also Microsoft Excel spreadsheets that contain the formulas and methodology to calculate HANPP for crops and timber. These are called “HANPP_crop_calculator” and “HANPP_timber_calculator.” To utilize the timber HANPP calculator sheet, the user inputs timber harvest data at the county level in units of cubic feet for roundwood and mill residues, as well as all removals, for both hardwood and softwood. To utilize the crop HANPP calculator, the user inputs crop harvest data in the indicated units, which conform to USDA-NASS Quick Stats listings. The stoichiometry formulas in the spreadsheet will output HANPP measurements for specific products for HANPP total, aboveground, belowground, used, and unused in both gCm^−2^yr^−1^ and tonnes.

In the repository there is also an R markdown file that contains code used to produce the HANPP bar charts that we included in the accompanying paper. This code can be modified to produce different county, state, or national HANPP bar charts. There is commentary in the document to help guide the user.

## 4 Experimental Design, Materials and Methods

To calculate total HANPP for each county, we used a detailed, bottom-up approach. This was done by calculating three subsets of HANPP (crops, timber, and grazing) on a product-specific basis and adding them together to get a total HANPP signature for each county. The Mendeley Data page includes crop and timber HANPP calculators that users can employ for years not included in the study.

### 4.1 Calculating Crop HANPP

We obtained crop yield data for each county for all four years (1997, 2002, 2007, 2012) from USDA-National Agricultural Statistics Service Quick Stats [2]. For counties that did not have available crop data we used the USDA-NASS Cropland Data Layer [3] to make estimates. We converted these measurements to HANPP using the formula for calculating the NPP harvested during crop production from data on crop yields from [4]. Note that correspondence with the authors showed that root:shoot ratio published in that paper was incorrect and was replaced with % shoot.

HANPP (harvest) = (economic yield * dry fraction *carbon content) / (harvest index * % shoot)

We developed crop-specific stoichiometry estimates from peer-reviewed, gray, and extension literature to estimate the different economic yield, dry fraction, carbon content, and harvest index percent shoot for each of the crops (Table 1). Economic yield is the area harvested (usually in acres) times yield per unit area (in bushels, pounds or tons). Dry fraction is the average percentage weight retained when water is driven off. Carbon content is 45% for all biomass [5]. Harvest index is the percentage of the aboveground biomass that constitutes yield of the product. Percent shoot is the proportion of crop biomass occurring above-, rather than below-ground. We identified the leading crops grown in the U.S. from the USDA-NASS Cropland Data Layer. The ten high-acreage major crops from Quick Stats data are corn grain and silage; winter, spring and durum wheat; alfalfa hay; pima and upland cotton; and sorghum. These main crops occupied 77 percent of cropland in 2012. Dry fraction varies from 82 to 93.5 percent for all crops except corn silage (35 percent). These calculations were done with Microsoft Excel software.

**Table 1:** Stoichiometry for converting crop yield and timber production to HANPP for each crop studied (Adapted from Tables 1 and 2 in [1]).

Product	Density (lb/ft^3^)	Dry Fraction	Carbon Content	% Shoot	Harvest Index
*Crop*					
Corn Grain	Na	0.845	0.45	0.85	0.53
Corn Silage	Na	0.350	0.45	0.85	1.00
Wheat	Na	0.865	0.45	0.83	0.45
Soybeans	Na	0.870	0.45	0.87	0.46
Alfalfa-Hay	Na	0.820	0.45	0.46	1.00
Cotton	Na	0.935	0.45	0.86	0.47
Sorghum	Na	0.880	0.45	0.86	0.47
*Timber*					
Softwood	31	0.75	0.45	0.79	na
Hardwood	43	0.78	0.45	0.80	na

In addition to calculating HANPP for the main crops, we also calculated HANPP of all minor crops aggregated together (we refer to them in the dataset as “Other Crops”). The Cropland Data Layer in 2012 was used with ArcGIS software to identify the acreage of minor crops in each county. However, data on yields and the stoichiometry of these numerous minor crops are not available. We therefore assumed that the on-site HANPP density of major crops (408gCm^−2^yr^−1^) is the national mean for minor crops. We also assumed this to be constant for 1997, 2002 and 2007 because Cropland Data Layer data only became available for the entire U.S. in 2008.

Using Microsoft Excel software, we used the stoichiometry formulas to calculate the NPP harvested for each crop in each county in each year studied. We then totaled all 10 major crops and Other Crops to calculate county HANPP measurements for all 3101 counties. We derived county-wide HANPP and onsite HANPP densities in gCm^−2^yr^−1^ and total mass of HANPP in kilotonnes C. HANPP was further divided into used and unused portions as determined by the harvest index and above- and below-ground portions as determined by percent shoot.

### 4.2 Calculating timber HANPP

Data on softwood and hardwood harvests (in thousand cubic feet) for each CONUS county in the years 1997, 2002, 2007 and 2012 were obtained from the U.S. Forest Service database Timber Product Output (TPO) [6] The timber data for each of these years represents an average yearly timber harvest calculated from measurements from the previous five years. The raw data include several types of timber products. Our study defines the product “roundwood” as used HANPP and all other removals (i.e., ‘slash’) as unused HANPP that remains at the harvesting site. We converted county-level TPO data from thousand cubic feet to HANPP using stoichiometry in Table 1 applied to the formula:

TimberHANPP=(allremovals*dryfraction*carboncontent)/(%shoot)

This produced timber HANPP measures for hardwood and softwood for each county in each year in total metric kilotonnes of carbon and county-average density in gCm^−2^yr^−1^. On-site densities could not be calculated because the data are not site-specific within the county and the U.S. Forest Service does not report the area harvested.

### 4.3 Calculating grazing HANPP

Estimating HANPP from livestock grazing is less straightforward than crops or timber because grazing takes place on both public and private lands that have quite different data sources. For public lands, the USDA Forest Service (USFS) and the Bureau of Land Management (BLM) provide permits to ranchers in the form of animal-unit months (AUMs) appurtenant to allotments of land [7]. Allotments were assigned to counties in proportion to the area that lies within each. We obtained data on AUMs authorized, which vary annually but never exceed the amount permitted. These data are provided periodically by the USFS and BLM Permit Schedule Information Reports. AUMs are conceptually identical to HANPP and are defined as the forage needed for a 1000-pound cow and her calf for one month (26 pounds of dry matter per day, which converts to 162 kgC of HANPP). We applied this metric to the AUM data we obtained to measure total metric tonnes of carbon and on-site densities in gCm^−2^yr^−1^ for each allotment in the most recent permit.

For private lands, we first quantified the NPP resources contained in grassland and pasture in each county. This was done by matching 30m pixels classified as grassland/pasture in the Cropland Data Layer with NPP data from Landsat in 2012. We then categorized counties into 20 USDA Land Resource Regions (LRR) that may vary in the percentage of NPP that is utilized by livestock. Within each LRR, counties were selected that had a population below 100,000 and lacked confined animal feedlot operations (CAFOs). We then quantified grazing demand by obtaining data on the number of beef cattle from the USDA-NASS Cattle Inventory for the years 1997, 2002, 2007, and 2012. Each beef cattle was assumed to require 12 AUMs or 1944 kgC per year from grazing. The total grazing demand for selected counties in each LRR was then compared to NPP resources in those counties to derive a percentage of NPP grazed as the measure of HANPP in each respective LRR (Table 2). These calculations were done using Microsoft Excel software.

**Table 2:** Proportion of NPP in grassland/pasture grazed in each Land Resource Region in 1997, 2002, 2007, 2012.

Land Resource Region	1997	2002	2007	2012
Atlantic Gulf Coast Lowland	0.120	0.136	0.129	0.105
Central Feed Grains	0.099	0.102	0.104	0.070
Central Great Plains	0.132	0.157	0.123	0.105
California	no data	0.082	0.077	0.070
East And Central Farming	0.168	0.163	0.167	0.140
Florida	0.181	0.154	0.159	0.159
Lake States	0.052	0.021	0.042	0.021
Mississippi Delta	0.080	0.077	0.092	0.081
Northern Great Plains	no data	0.072	0.066	0.071
Northern Lake States	0.063	0.055	0.020	0.054
North Atlantic	0.227	0.200	0.174	0.186
Northeast	0.115	0.090	0.115	0.044
Northwest Forest	0.051	0.048	0.081	0.040
Northwest Wheat	0.178	0.184	0.178	0.161
Rocky Mountain	0.178	0.109	0.081	0.101
South Atlantic And Gulf Slope	0.147	0.135	0.133	0.119
Southwest Plateaus	0.267	0.256	0.234	0.194
Southwest Prairies	0.165	0.160	0.153	0.136
Western Great Plains	0.061	0.061	0.057	0.051
West Range	0.132	0.165	0.126	0.169

Total grazing HANPP in each county was divided by the area of grassland/pasture to obtain on-site density and by the area of the county to obtain county-wide HANPP density in gCm^−2^yr^−1^. Grazing HANPP was defined as entirely used and entirely above-ground.

### 4.4 Calculating total HANPP

All crop, timber, and grazing HANPP was summed to get a measurement of total HANPP for the 3101 CONUS counties. This was then summed to get a measurement of national HANPP. National HANPP totals are in the units of total metric kilotonnes of carbon and densities are measured in gCm^−2^yr^−1^. HANPP in kilotonnes was calculated by summing all HANPP totals, while HANPP in gCm^−2^yr^−1^ was calculated by summing all HANPP in gC but dividing by the total area of all counties in m^2^. These calculations were done using Microsoft Excel software.

### 4.5 Calculating NPP

The net primary production of each county was calculated by downloading Moderate Resolution Image Spectroradiometer (MODIS) data [8], converting all pixel values to gCm^−2^yr^−1^ and aggregating 250m pixels for each county. NPP(ecological) was calculated by subtracting total HANPP(harvest) from each county from NPP in terms of kilotonnes and county-wide density in gCm^−2^ yr^−1^.

## Ethics Statements

The authors have read and followed the ethical requirements for publication in *Data in Brief*. Neither human study participants, animals, nor social media platforms were involved in collecting the data presented here. All data used in the study are public domain.

## CRediT authorship contribution statement

**Suman Paudel:** Methodology, Software, Validation, Formal analysis, Investigation, Data curation, Visualization. **Kaeli Mueller:** Software, Validation, Formal analysis, Data curation, Writing – original draft, Visualization. **Gustavo Ovando-Montejo:** Methodology, Software, Validation, Formal analysis, Investigation, Data curation, Visualization. **Lauren Tango:** Methodology, Software, Validation, Formal analysis, Data curation. **Richard Rushforth:** Conceptualization, Methodology. **Christopher Lant:** Conceptualization, Methodology, Validation, Formal analysis, Investigation, Writing – review & editing, Supervision, Project administration, Funding acquisition.

## Data Availability

A Dataset Cataloging Product-Specific Human Appropriation of Net Primary Production (HANPP) in US Counties (Original data) (Mendeley Data).
